# Fetal brain volumes and brain gyrification index associated with opioid exposure

**DOI:** 10.1093/braincomms/fcag158

**Published:** 2026-04-30

**Authors:** Ramana V Vishnubhotla, Jonathan A Class, Yi Zhao, Lindsey L Zelt, William T Reynolds, David M Haas, Sanjali Kherde, Senthilkumar Sadhasivam, Rupa Radhakrishnan

**Affiliations:** Department of Radiology and Imaging Sciences, Indiana University School of Medicine, Indianapolis, IN 46202, USA; Department of Radiology, University of Pittsburgh School of Medicine, Pittsburgh, PA 15213, USA; Department of Radiology and Imaging Sciences, Indiana University School of Medicine, Indianapolis, IN 46202, USA; Department of Biostatistics and Health Data Science, Indiana University School of Medicine, Indianapolis, IN 46202, USA; Department of Radiology and Imaging Sciences, Indiana University School of Medicine, Indianapolis, IN 46202, USA; Department of Radiology, University of Pittsburgh School of Medicine, Pittsburgh, PA 15213, USA; Department of Obstetrics and Gynecology, Indiana University School of Medicine, Indianapolis, IN 46202, USA; Purdue University, Indianapolis, IN 46202, USA; Department of Anesthesiology and Perioperative Medicine, University of Pittsburgh School of Medicine, Pittsburgh, PA 15261, USA; Department of Radiology and Imaging Sciences, Indiana University School of Medicine, Indianapolis, IN 46202, USA; Department of Radiology, University of Pittsburgh School of Medicine, Pittsburgh, PA 15213, USA

**Keywords:** prenatal opioid exposure, fetal MRI, fetal brain volume, NOWS, fetal brain reconstruction

## Abstract

Opioid use during pregnancy continues to be a significant public health challenge, with antenatal opioid exposure suggesting adverse infant and child health outcomes. Early identification of risk of future neurodevelopmental disorders could help improve long-term health outcomes in these children. In this study, we assess whether prenatal opioid exposure is associated with early alterations in human fetal brain development identifiable on fetal MRI. This was a prospective, institutional review board approved, multicenter study that recruited pregnant women on medication-assisted treatment (MAT) for opioid use disorder (OUD) and a control cohort of non-OUD pregnant women. Fetal brain MRI was performed in the second or third trimester of gestation. Semiautomated pipelines were used to create super-resolution 3D isotropic anatomic brain magnetic resonance images from 2D fetal brain images and segmented into different tissue types. Fetal brain gyrification indices were also calculated. Differences between fetuses with opioid exposure and control fetuses were assessed. There were 63 fetuses in this study—31 with opioid exposure and 32 without opioid exposure (controls). Fetuses with opioid exposure had significantly smaller fetal brain total volumes, fetal brain white matter volume, and significantly lower fetal brain gyrification index compared to control fetuses. In opioid exposed fetuses, fetal brain volumetric and surface-based metrics did not correlate with severity of neonatal opioid withdrawal syndrome (NOWS) requiring longer hospitalization or postnatal opioid medication treatment. Additional and larger prospective studies are needed to understand how fetal brain development may be associated with short- and long-term outcomes.

## Teaser

Opioid use during pregnancy can lead to poor neurodevelopment. This study assesses the association of maternal opioid use with fetal brain development. Maternal opioid use was significantly associated with lower fetal brain volumes, white matter volumes and gyrification indices. This highlights impaired brain development prior to birth.

## Graphical Abstract

**Graphical Abstract:**
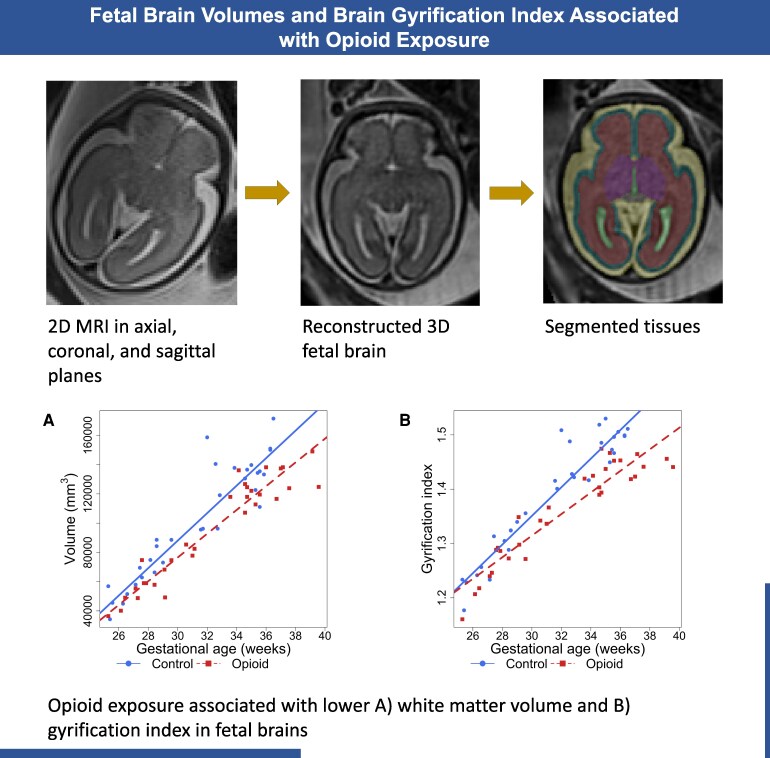

## Introduction

Opioid use in pregnancy continues to rise in the USA.^1,2^ From a 2019 survey, 6.6% of women reported opioid use during pregnancy. Of women indicating opioid use, 21.2% reported misuse, 27.1% indicated a desire to reduce usage, and 31.9% did not receive proper guidance on the impact of opioid use on the infant.^3^ The effects of prenatal opioid exposure (POE) on child development range from adverse birth outcomes like lower birthweight, smaller head circumference and neonatal opioid withdrawal syndrome (NOWS) to correlations of longer-term outcomes, notably neurocognitive delay.^4–7^ Potential mechanisms for these associations have been researched in preclinical studies.

Many studies have correlated POE with neurocognitive and child developmental delays.^6–8^ For example, POE was associated with worse motor and visual outcomes.^6,9,10^ Receptive and expressive language was also shown to be affected in a cohort of four-year-old children.^11^ MRI morphologic studies in children with POE have shown regional brain differences despite no significant differences in global brain sizes.^12–14^

Endogenous opioid receptors play an important role in healthy CNS development and function.^15^ Therefore, exogenous opioids introduced in pregnancy may contribute to altered maturation of the developing brain and hence adverse short- and longer-term neurodevelopment. Buprenorphine and methadone are two most recommended medications used in medication-assisted treatment (MAT) in pregnancy to manage opioid use disorder (OUD). These medications curb symptoms of craving and withdrawal and decrease fetal distress, also decreasing incidence and severity of postnatal opioid withdrawal.^16,17^

Several studies have shown altered postnatal brain structure and function in POE, including smaller brain volumes in the deep grey structures, regional changes in white matter volumes, smaller CSF volumes and altered white matter microstructure compared to non-opioid-exposed infants.^14,18^ Researchers, including our group, have also demonstrated alterations in postnatal infant brain functional connectivity in POE,^19,20^ that correlate with severity of NOWS.^21^ Despite in vitro and animal studies showing altered early myelination and neuronal impairment when exposed to opioid maintenance therapies, methadone and buprenorphine,^22–24^ a human study suggests some benefit from MAT in pregnancy to partially normalize the effects of prenatal drug exposure on developing brain functional connectivity in infancy.^25^ Disentangling the effects of OUD, polysubstance exposure and associated medical treatments is challenging, and further research is required in this area.

Other confounders in primarily postnatal studies evaluating effects of prenatal exposures are birth-related and perinatal events that may potentially impact accurate assessment of these prenatal exposure-related effects. Here, fetal imaging adds value in the assessment of the effects of substances on the developing brain prenatally. We and other researchers have shown alterations in linear brain metrics and white matter structure in fetuses with POE and smoking exposures using fetal MRI.^26–28^ A single small study of 14 opioid-exposed fetuses suggested altered global brain volumes and brain surface metrics on third-trimester fetal MRI.^29^ However, based on postnatal studies, opioids may differentially impact the development of different regions of the brain, and it is not known whether differential brain development in POE may be evident in the fetal period.

To better understand the effects of POE on the developing brain, we conducted a prospective study using fetal MRI in second- and third-trimester fetuses in POE and control fetuses without exposure to opioids and other substances to assess the impacts of opioids and other prenatal substance exposures on fetal brain global and regional segmented volumes and surface brain metrics (gyrification index).

## Materials and methods

### Study design and subjects

This was a prospective, IRB-approved, HIPAA-compliant multicenter study that recruited pregnant women with OUD on MAT and control healthy pregnant women. Subjects were recruited at the Indiana University School of Medicine and the University of Pittsburgh Medical Center. Informed consent was obtained according to the Declaration of Helsinki.^30^ Inclusion criteria for the pregnant women with opioid use were age 18 years or older, currently on MAT for OUD and singleton pregnancy. Inclusion criteria for the control group were women 18 years or older without prenatal substance exposure, and with a healthy singleton pregnancy. Control pregnant women were recruited from the same sites as the women on MAT and are reflective of a similar geographical cohort. Exclusion criteria included MRI contraindications, serious maternal medical illness and major fetal congenital abnormalities. Severe NOWS was determined by a neonate’s need for opioid medication treatment based on clinical algorithms and hospital length of stay (LOS). Demographic information and clinical data including details of opioid and polysubstance use, were obtained via detailed patient interview and medical records review. All data were electronically stored on REDCap.^31,32^

### MRI acquisition

Fetal brain MRI was performed in the late second or third trimester and fetal MRIs (25–38 weeks gestation). At the Indiana University (IU) site, all subjects underwent the same imaging protocol on a 3T Siemens Skyra scanner or 3T Siemens Vida Fit scanner (Erlangen, Germany) with a 16-channel body coil. The subjects at the University of Pittsburgh (UPitt) site underwent the same imaging protocol on a 3T Siemens Skyra scanner (Erlangen, Germany). Women were imaged in the lateral decubitus or supine positions. Both sites used a phased array body imaging coil for image acquisition. Two-dimensional MR images of fetal brains were acquired using 2-dimensional Half-Fourier Acquisition Single-Shot Turbo Spin Echo (HASTE) sequence in the axial, coronal and sagittal planes for all subjects. Assessed planes were based on fetal brain orientation. Acquisition parameters for both sites were TE = 78.00 ms, TR = 1100.0 ms, 46 slices, 3.0 mm slice thickness, resolution = 256 × 230, and total acquisition time = 52 s. If fetal motion affected quality, the MR technologists acquired multiple images in that plane. All images were sent to IU for analysis.

### Fetal brain 3D reconstruction

Acquired 2D HASTE sequences were visually inspected for quality. Individual plane images were excluded when significant artefacts were present. An automated segmentation pipeline, MONAIfbs,^33^ was used to create fetal brain mask for each 2D image and the outputs were visually assessed for quality. MONAIfbs is a fetal brain segmentation tool which takes advantage of the PyTorch-based Medical Open Network for Artificial Intelligence (MONAI) framework, based on the dynamic UNet (dynUNet), an adaptation of the nnU-Net framework.^33^

**Figure 1:**
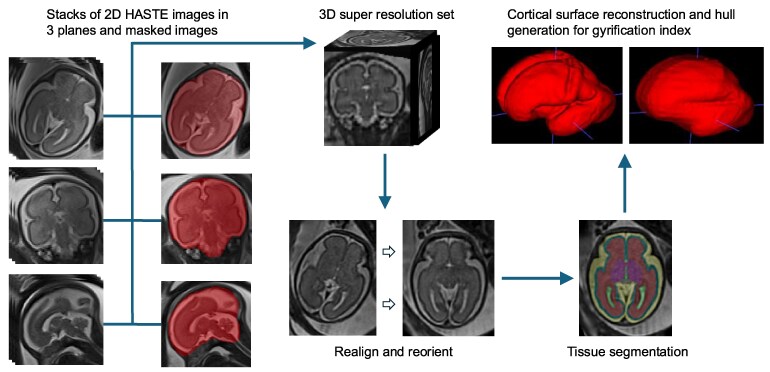
**Workflow for fetal brain segmentation and calculation of gyrification index.** Fetal brains were segmented from 2D MRI images in all three planes. Images from all planes plus their respective masks were used to create the 3D super-resolution reconstructions. Output files were aligned to template space and then run through a trustworthy AI fetal brain tissue segmentation pipeline. Gyrification index was calculated based on the pial surface from the segmentation.

We used ITK-Snap,^34^ a free, open-source, multi-platform software application that can segment structures in 3D and 4D biomedical images for minor manual correction to the segmentation. NiftyMIC, a Python-based open-source toolkit was used to create 3D isotropic super resolution reconstructions (0.8 mm isotropic) from 2D HASTE images and corresponding masks by employing slice-to-volume registration algorithms to correct for fetal motion and align the individual 2D slices to create a consistent 3D volume.^35^ The output super resolution 3D reconstructions were re-oriented into standard planes and manually re-registered to a gestational age-appropriate fetal brain template provided by Gholipour *et al*.^36^ using ITK-Snap. This was a rigid-body transformation and did not alter fetal brain morphology. All 3D reconstructions were further rigorously checked for quality by a paediatric neuroradiologist and any images with any substantial artefacts that affected even a single slice were discarded. Fetal brain analysis workflow is depicted in Fig. 1.

### Fetal brain analysis

After quality inspection, the 3D super resolution reconstructions of the fetal brain were segmented using a validated trustworthy AI framework^37^ based on the Dempster–Shafer approach to segment fetal brain images. As described by Fidon *et al*., this framework consists of a backbone AI algorithm for the task at hand, a fallback segmentation algorithm that is more robust than the backbone AI algorithm to out-of-distribution data (although potentially less precise), and finally, a fail-safe method that automatically detects local conflicts between the backbone AI algorithm prediction and the contracts of trust and switches to the fallback algorithm in case of conflicts. For this specific application, the principled fail-safe method is based on Dempster-Shafer theory.^37^

Using this approach, we segmented 8 tissue types—white matter (includes intermediate zone and subplate zone), ventricles, cerebellum, cerebrospinal fluid outside the ventricles (CSF), cortical plate, deep grey (includes thalamus, basal ganglia and germinal matrix), brainstem, and corpus callosum and regional volumes were extracted. A paediatric neuroradiologist visually inspected all 3D segmentations for accuracy. To calculate brain gyrification index, the cerebellum, CSF and brainstem were removed from the segmented 3D images to display the pial surface. Using MATLAB, an outer convex hull volume and surface were created based on the trimmed segmented pial surface. The gyrification index was calculated by taking the ratio of the pial surface area to the outer hull surface area as performed previously.^38^ Code for gyrification index can be found in the Supplementary material (gyrification_index.m).

### Statistical analysis

We used multiple linear regression analysis to assess group differences for each imaging feature between the opioid exposed and control fetuses. The model included brain imaging metrics (whole brain volumes and gyrification index, and regional brain volumes of fetal white matter, ventricles, cerebellum, CSF, cortical plate, deep grey, brainstem and corpus callosum) as the response variable and controlled for race, fetal sex, gestational age, tobacco exposure and polysubstance. In fetuses with opioid exposure that had postnatal clinical NOWS data available, we used linear models to test for associations of fetal brain metrics with postnatal metrics of NOWS severity—need for postnatal opioid medication and LOS. Race, fetal sex, gestational age, maternal tobacco exposure and polysubstance use were included as covariates in the model. Multiple comparison correction to assess false discovery rate (FDR) was applied to both sets of analyses of volumetric data using the Benjamini–Hochberg procedure.^39^ An FDR less than 0.05 was considered significant.

## Results

### Demographics and clinical data

There were 200 opioid-using pregnant women and 622 healthy pregnant controls approached for this study. Of these, there were 116 from the opioid group and 145 from the control group who enrolled in the study. In the recruitment phase, there were 13 from the opioid group and 27 from the control group who did not meet the inclusion criteria or withdrew from the study. Fetal MRI containing 2D HASTE images were available from 95 subjects. Of these, 32 subjects were excluded for poor scan quality of initial HASTE images, poor quality of 3D reconstruction, and MRI outside gestational window. Of the 63 fetuses included in the final analysis, there were 31 with opioid exposure and 32 unexposed controls. In the opioid-exposed group, postnatal NOWS-related clinical outcomes were available for 29 (94%) of the 31 subjects. For these 29 infants the median length of hospital stay was 5 days (range 4–42 days). The demographic and clinical data are summarized in Table 1. Data of the excluded 32 subjects are included in the Supplementary Table 1 (Supplementary_Table1.docx).

**Table 1:** Demographic and clinical data

	Opioid	Control	*P*-value
Number	31	32	
Sex (male)	18	15	0.453^a^
Gestational age in weeks (SD)	32.25 (4.3)	31.31 (3.8)	0.363^b^
Maternal age in years (SD)	31.32 (5.3)	29.69 (4.9)	0.211^b^
Tobacco exposure	23	0	<0.001*,^a^
Polysubstance exposure	12	0	<0.001*,^a^
Alcohol	1	0	
Cocaine	1	0	
Fentanyl	5	0	
Heroin	2	0	
LSD	1	0	
Marijuana	7	0	
Methamphetamine	1	0	
Oxycodone	1	0	
Race			<0.001*,^a^
White	26	18	
Black	0	13	
Asian	1	0	
Mixed/other	4	1	
Scanner			0.493^a^
IU1	12	17	
IU2	9	6	
Pitt	10	9	
Opioid medication-assisted treatment			
Methadone	7	N/A	
Buprenorphine	24	N/A	
Severity of NOWS			
Need for postnatal opioid treatment (29 neonates with available data)	7	N/A	
Hospital length of stay in days (SD)	10.34 (10.1)	2.16 (1.1)	<0.001*,^b^

^*^Denotes significance.

^a^Fisher’s exact test.

^b^Independent *t*-test assuming unequal variances.

### Fetal brain global and regional metrics

**Figure 2:**
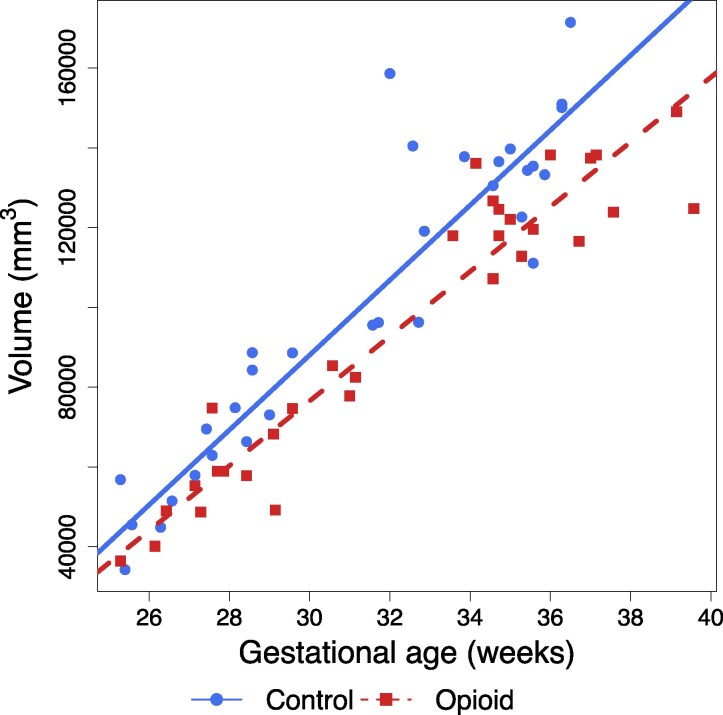
**Association of white matter volume and prenatal opioid exposure.** A linear regression was performed to assess the association of opioid exposure and white matter volume (*n* = 63; 31 with opioid exposure, 32 controls; *R*^2^ = 0.975). Scatter plot of fetal brain white matter volume (mm^3^) corrected for sex, race, tobacco, and polysubstance use versus gestational age (weeks) in opioid-exposed and unexposed fetuses. Opioid exposure was significantly associated with reduced white matter volume (*t* = −3.09; *P* = 0.003). A false discovery rate < 0.05 was considered significant.

**Figure 3:**
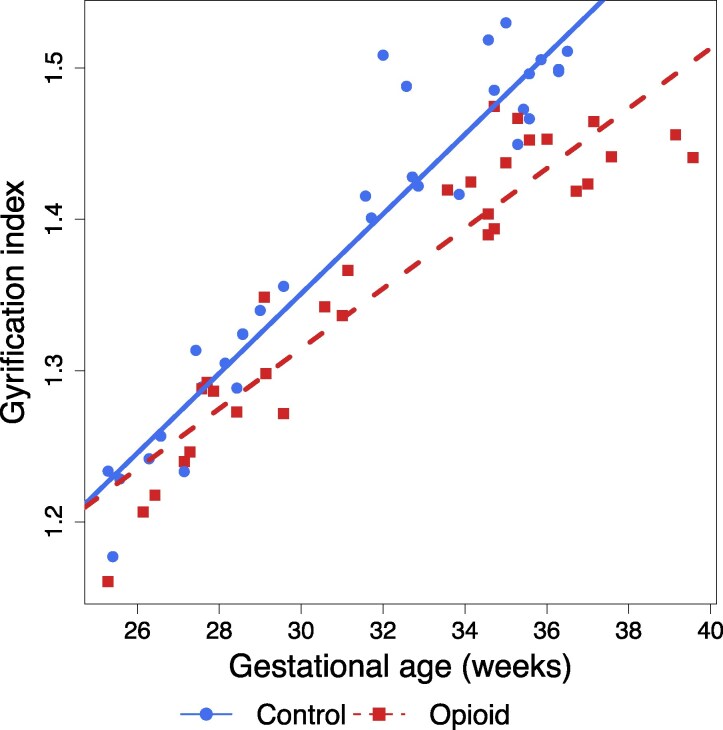
**Association of gyrification index and prenatal opioid exposure.** A linear regression was performed to assess the association of opioid exposure and gyrification index (*n* = 63; 31 with opioid exposure, 32 controls; *R*^2^ = 0.923). Scatter plot of corrected gyrification index (ratio) corrected for sex, race, tobacco, and polysubstance use versus gestational age (weeks) for opioid-exposed and unexposed fetuses. Opioid exposure was significantly associated with a reduced gyrification index (*t* = −3.28; *P* = 0.002). A *P*-value < 0.05 was considered significant.

Global brain volumes were significantly smaller in opioid-exposed fetuses compared to controls after correcting for multiple comparisons (*t* = −2.67; *P* = 0.01). Fetal white matter, deep grey matter and corpus callosum volumes were significantly smaller in opioid-exposed fetuses compared to controls on individual analyses. After multiple comparisons corrections, only fetal white matter volume was significantly different between the two groups. (Table 2, Fig. 2) Based on a linear model, fetal brain gyrification index was significantly lower (*t* = −3.28; *P* = 0.002) in fetuses exposed to opioids than unexposed control fetuses (Fig. 3). There was a trend towards a larger difference in gyrification indices between the opioid-exposed and unexposed fetuses with increasing gestational age; however, this did not reach statistical significance (*P* = 0.0675, Fig. 3). There were no significant associations between fetal brain metrics and the need for postnatal opioid treatment in the neonate or length of hospital stay.

**Table 2:** Linear regressions were performed for each segmented brain volume with demographic and exposure related covariates

Fetal brain regions	*t*-stat	Uncorrected *P*-value	FDR
White matter	−3.09	0.003	0.025*
Ventricles	−1.25	0.217	0.217
Cerebellum	−1.67	0.100	0.160
CSF	−1.40	0.166	0.190
Cortical plate	−1.81	0.076	0.152
Deep grey	−2.34	0.023	0.092
Brainstem	−1.50	0.140	0.187
Corpus callosum	−2.17	0.035	0.092

After multiple comparison correction, a false discovery rate (FDR) < 0.05 was considered significant.

^*^Denotes statistical significance after multiple comparison correction.

## Discussion

Opioid use during pregnancy continues to be a serious problem which has been linked to numerous childhood developmental neurobehavioral disorders.^6,7,40^ This study, which assessed the association of prenatal maternal opioid exposure with human fetal brain global and regional volumes and surface metrics, demonstrates for the first time to our knowledge, significantly lower fetal brain white matter with rigorous multiple comparison correction. We also identified a significantly lower fetal brain gyrification index in the setting of opioid exposure with multiple comparison corrections. In addition, smaller deep grey matter and corpus callosum volumes in fetal brain were also associated with POE on individual analyses; however, with multiple comparison correction, these associations were not significant.

Our study used robust semiautomated pipelines to produce super-resolution 3D fetal brain images from stacks of 2D HASTE images acquired in the axial, coronal and sagittal planes.^33,35^ We were also able to effectively segment the reconstructed 3D brain into regional volumes consisting of the most common tissues using an automated pipeline.^37^ The workflow presented in this study could be utilized for wide range of applications in fetal brain imaging. We did not use fetal imaging prior to 25 weeks gestation due to the dominant multilaminar fetal MRI pattern during the midportion of the second trimester that may impact segmentations.

Global and specific regional brain volumes are shown to be smaller in infants and children prenatally exposed to opioids including in the deep grey matter, brainstem, cerebrospinal fluid and subinsular white matter.^14^ Paradoxically, certain brain regions such as white matter in the right cingulate gyrus and left occipital white matter had larger volumes.^14^ Smaller global and regional brain volumes including in the amygdala, basal ganglia and corpus callosum as well as reduced regional cortical surface areas persist until later childhood and adolescence in prenatal exposure to opioid maintenance therapy.^12^ Similar to these postnatal studies, we identified significantly smaller global brain volumes in fetuses with opioid exposure. We also noted a trend towards smaller deep grey volumes in opioid exposed fetuses (significant on individual comparison, but not with multiple comparison correction). There is rapid growth and development of the deep grey structures in the second trimester,^41^ and the trend towards lower volumes identified in our study suggests an early developmental impact of opioid exposure on the proliferating and migrating neurons.

We also showed significantly decreased fetal white matter volume (comprising the intermediate zone and subplate zone) in fetuses with opioid exposure compared to control fetuses. The intermediate and subplate zones are vital and dynamic regions in fetal brain development. The intermediate zone contains the migrating neurons in an astrocytic scaffolding that eventually develops into white matter in adults, and the subplate region situated just below the cortical plate contains many of the early maturing neurons, as well as neuronal projections and astrocyte precursors.^42^ Reduced volumes in these regions may be reflective of altered neuronal and glial proliferation and differentiation with opioid exposure in utero identified in preclinical studies.^43^ Indeed, a postnatal study in neonates with POE showed by Wu *et al*.^44^ revealed altered brain volumes in cortical and subcortical regions that may reflect downstream effects of lowered volumes in neuronal precursor regions seen in our study. A previous study showed higher fractional anisotropy (FA) in certain fetal white matter regions in the third trimester compared to control fetuses without exposure to opioids.^28^ Since much of the brain is unmyelinated in the fetal period, this alteration in FA values prenatally could potentially be related to alterations in neuronal migration and astroglial organization, or as previously hypothesized, related to a proinflammatory milieu in opioid exposure.^45^ Postnatal alterations in neonatal white matter microstructure have been seen in infancy with altered FA values and altered structural connectivity,^18,46^ that appear to persist to childhood^47^ and may indicate longer-term sequela of altered fetal brain development with opioid exposure.

Gyrification is an important process of development as it allows for greater surface area for neuronal connections. Developmentally, this process accelerates in the third trimester of pregnancy.^48,49^ This is also consistent with the data we observed in our study, as there was a trend towards a greater difference in the gyrification index between opioid-exposed and unexposed fetuses with increasing gestational age. As gyrification has been positively associated with cognitive abilities,^50^ impaired gyrification during the third trimester of pregnancy may partly explain impaired long-term neurodevelopmental and neurocognitive outcomes in children with POE.^7^ The results of our study add further detail to the previous study by Yun *et al*. showing smaller surface area, sulcal depth, and mean curvature in the third trimester in opioid-exposed fetuses.^29^ Interestingly, we could not replicate the previous association of cortical plate volumes and POE that was identified by Yun *et al*., likely because our fetal cohort were of younger gestational age (as young as 25 weeks postmenstrual age) compared to the previous study, which only included third trimester fetuses. Given the rapid neuronal and glial proliferation, migration and cortical organization that occur in the late second and third trimesters, the effects of opioids on brain regional volumes are likely to be much more striking later in gestation.

Similarly, although we identified smaller corpus callosal volumes in opioid-exposed fetuses on individual regression analysis, this did not withstand multiple comparison correction, likely due to the varied developmental trajectories of the different fetal brain regions. Reduced corpus callosal length has been reported on prior 2D fetal imaging,^27^ and reduced FA in the corpus callosum with opioid exposure is reported in animal studies.^51,52^

The benefit of fetal imaging is to understand early developmental impacts of prenatal exposures and their utility in prediction of clinical outcomes. Fetal imaging reduces biases such as infant feeding and neonatal intensive care unit stay that are present in neonatal and infant studies. It also allows for earlier risk assessment, potentially improving the timing and effectiveness of treatments. We identified different associations of late second- and third-trimester fetal brain volumes and gyrification with opioid exposures that likely reflect the different developmental trajectories of the fetal brain regions that we segmented. In opioid-exposed fetuses, we did not identify an association with short-term outcomes of severity of NOWS. This may reflect the multifactorial aetiology of NOWS, which may be better assessed through measurements of neuronal function identified on brain functional connectivity.^21^ We also only had a small sample size of seven neonates with POE requiring postnatal opioid treatment.

There were a few limitations to our study. First, fetal imaging is challenging due to fetal motion and image quality degradation. However, all images were visually inspected at multiple stages in our processing pipeline to ensure accuracy. While we have a relatively large sample size for fetal imaging, this is still a limited sample to include other variables of interest. Only six subjects in the opioid-exposed cohort had severe postnatal NOWS requiring medication treatment, and likely too small for us to find meaningful associations on prenatal brain imaging. Finally, similar to what has been reported by other studies, many of the pregnant women with opioid exposure often had tobacco and polysubstance exposure, making it challenging to isolate potential opioid effects. To account for this, we controlled for tobacco and polysubstance use in our model; however, these additional exposures are representative of the population we are studying. Despite our limitations, our study provides new insight into the associations of opioid exposure in pregnancy and the developing fetal brain.

## Conclusion

This study identified novel and significantly lower fetal white matter volumes, smaller total brain volumes and lower gyrification indices in human fetuses with POE compared to control infants. In infants with POE, fetal brain volumetric and surface-based assessments did not correlate with severe NOWS needing medical interventions and longer hospitalization in the current study. Additional and larger prospective future studies assessing the utility of fetal brain imaging changes predictive of severe NOWS and longer neurodevelopmental trajectory of these infants are needed.

## Supplementary Material

fcag158_Supplementary_Data

## Data Availability

The data that support the findings of this study are available from the corresponding author and ethical board approval upon reasonable request. Relevant code is available in the Supplementary material.
