# Supplementary material for: Fetal brain volumes and brain gyrification index associated with opioid exposure
Source: Brain Commun. 2026 Apr 30;8(3):fcag158. doi: 10.1093/braincomms/fcag158 (PMC13184692; doi:10.1093/braincomms/fcag158)
Supplement: fcag158_Supplementary_Data [file fcag158_supplementary_data.zip › Supplementary_Table_1.docx]

**Supplementary Table 1:** Demographic and clinical data of excluded subjects.

|  | **Opioid** | **Control** | **p-value** |
| --- | --- | --- | --- |
| Number | 16 | 16 |  |
| Sex (male) | 6 | 3 | 0.433^^ |
| Gestational age in weeks (SD) | 28.66 (5.5) | 29.79 (6.3) | 0.593^ |
| Maternal age in years (SD) | 31.88 (5) | 28.87 (4.9) | 0.096^ |
| Tobacco exposure | 13 | 1 | < 0.01*^^ |
| Polysubstance exposure | 8 | 0 | < 0.01*^^ |
| Alcohol | 1 |  |  |
| Cocaine | 2 |  |  |
| Fentanyl | 2 |  |  |
| Heroin | 3 |  |  |
| Benzodiazepines | 2 |  |  |
| Marijuana | 6 |  |  |
| Methamphetamine | 3 |  |  |
| Oxycodone | 1 |  |  |
| Race |  |  | 0.362^^ |
| White | 14 | 10 |  |
| Black | 1 | 3 |  |
| Asian | 0 | 2 |  |
| Mixed/other | 1 | 1 |  |
| Scanner |  |  | 0.768^^ |
| IU1 | 4 | 5 |  |
| IU2 | 3 | 1 |  |
| Pitt | 9 | 10 |  |
| Opioid Medication Assisted Treatment |  |  |  |
| Methadone | 2 | N/A |  |
| Buprenorphine | 13 | N/A |  |
| Unknown | 1 | N/A |  |
| Severity of NOWS |  |  |  |
| Need for postnatal opioid treatment | 2 | N/A |  |
| Hospital length of stay in days (SD) | 9.4 (7.9) | 2.5 (2.2) | < 0.01*^ |

* Denotes significance

^ Independent t-test assuming unequal variances

^^ Fisher’s exact test
